# Correction: Extended poly(A) tails are a shared feature of herpesvirus mRNAs

**DOI:** 10.1371/journal.ppat.1014584

**Published:** 2026-09-14

**Authors:** Erik Fuhrmann, Sae Toda, Jonas Leins, Pierina Cetraro, Vedang Deshpande, Jasmine Rowell, Edward A. Chapman, Carina Jacobsen, Kai A. Kropp, Mart M. Lamers, Elene Loliashvili, Mostafa Saleban, Ruth Verstraten, Carolin Vogt, Wiyada Wongwiwat, Werner J. D. Ouwendijk, Abel Viejo-Borbolla, Robert E. White, Angus C. Wilson, Hannah M. Burgess, Daniel P. Depledge

Author Jonas Leins was incorrectly noted as a corresponding author. The correct corresponding authors are Hannah M. Burgess and Daniel P. Depledge. Hannah M. Burgess’s email address is: h.burgess@imperial.ac.uk.
