# Supplementary figures and images for: Extended poly(A) tails are a shared feature of herpesvirus mRNAs
Source: PLoS Pathog. 2026 Jun 16;22(6):e1014341. doi: 10.1371/journal.ppat.1014341 (PMC13271517; doi:10.1371/journal.ppat.1014341)

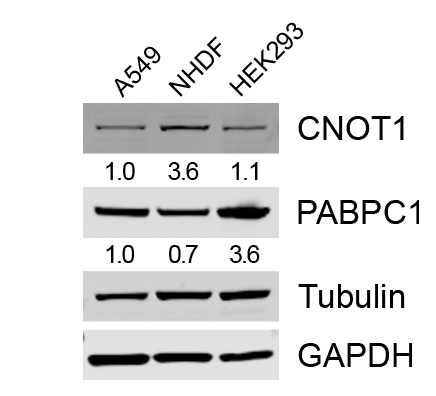

Supplement: S1 Fig — Total protein lysate (10 µg) from each of A549, NHDF and HEK293 cells was subjected to immune blotting for CNOT1, PABPC1 and two housekeeping proteins, GAPDH and alpha tubulin. Quantification of CNOT1 and PABPC1 abundance relative to that in A549 cells is indicated. (TIF) [file ppat.1014341.s001.tif]

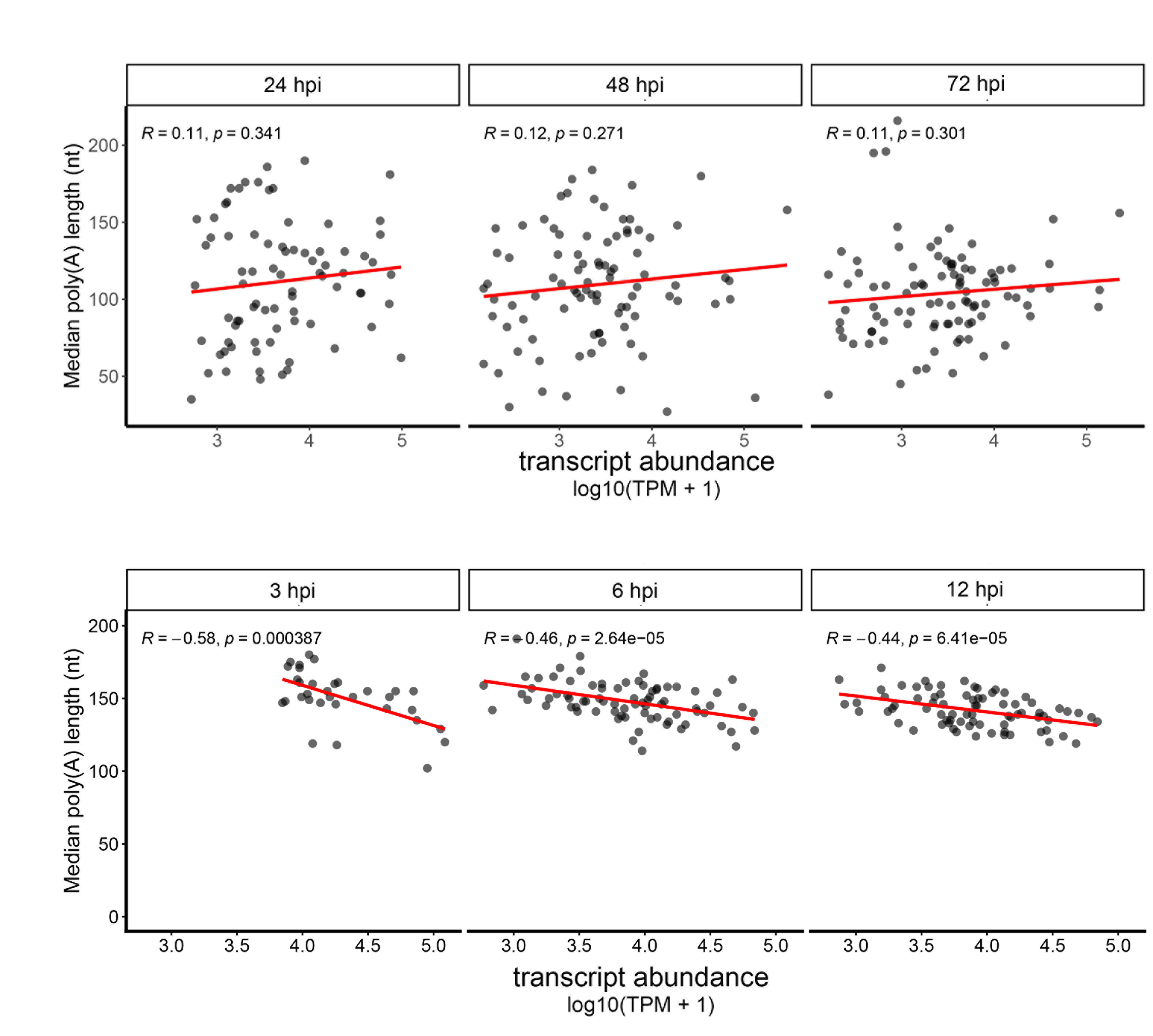

Supplement: S2 Fig — Scatter plots showing the relationship between transcript abundance and median poly(A) tail length at different time points post-infection (hpi) of NHDFs for (a) HCMV, and (b) HSV-1 mRNAs. Transcript abundance was normalised as transcripts per million (TPM) within each timepoint and log-transformed (log₁₀ (TPM + 1)) for visualisation. Each point represents an individual transcript. Red lines indicate linear regression fits. Pearson correlation coefficients (r) and associated p-values are shown for each timepoint. (TIF) [file ppat.1014341.s002.tif]

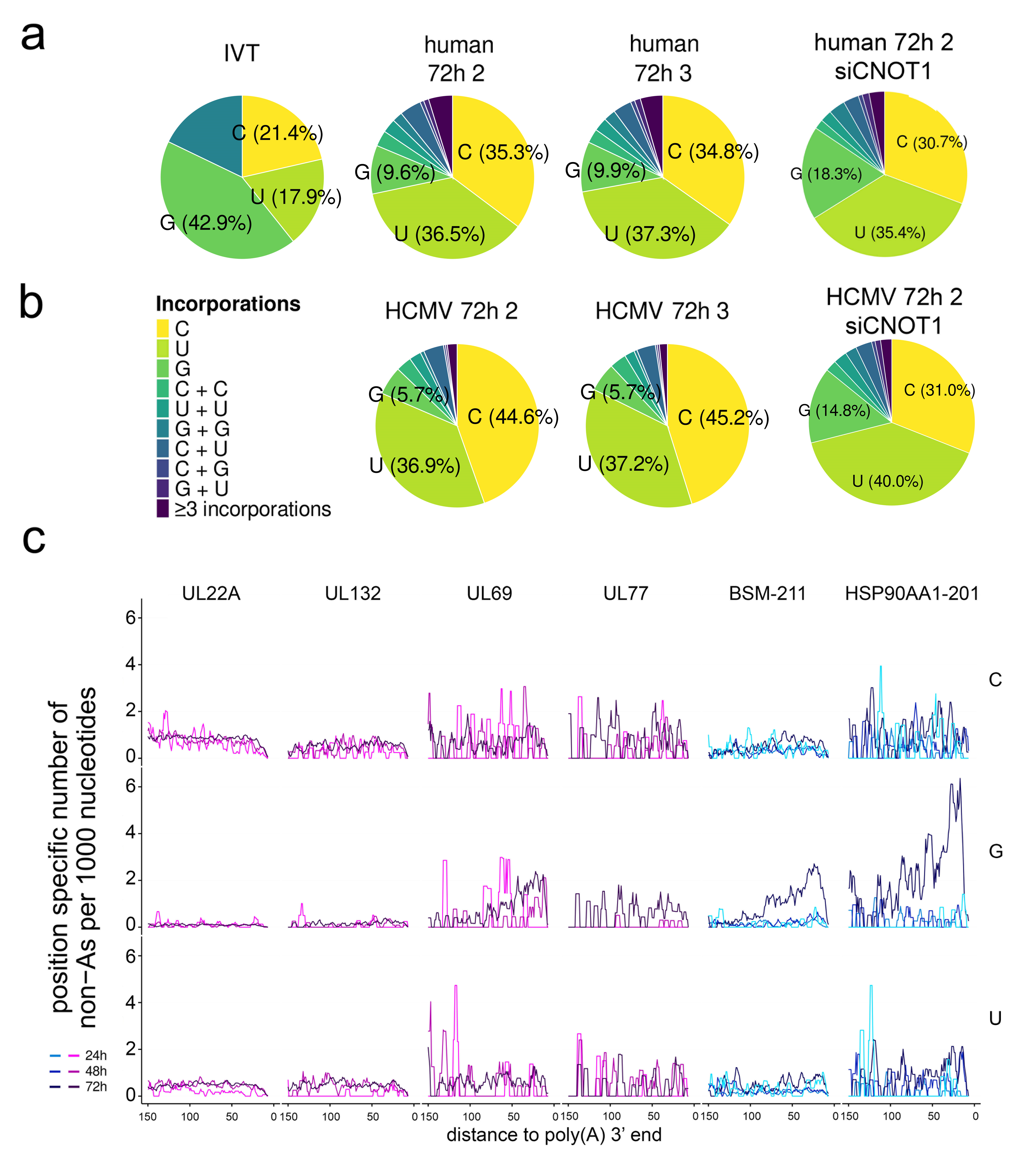

Supplement: S3 Fig — (a) Breakdown of nucleotides present in mixed tails for a synthetic RN7SK RNA with a clean tail and two additional biological replicates each for HCMV-infected NHDFs harvested at 72 hours post infection in either the presence or absence of siCNOT1 treatments. (a) cellular mRNA and (b) HCMV mRNAs. (b) Breakdown of nucleotides present in mixed tails of two additional biological replicates HCMV-infected NHDFs harvested at 72 hours post infection. (c) Position specific frequency of different non-A nucleotides in the poly(A) tail of selected transcripts and timepoints. Poly(A) tails have been aligned by their 3’ end, positions are defined by their distance of the ultimate 3’ nucleotide. The three 72h datasets have been pooled. Plotted frequencies are calculated by the number of specific non-adenosine residues with a certain distance d to the 3’ end relative to the total number of reads with a poly(A) tail length ≥d, averaged with a sliding window of five nucleotides. (TIF) [file ppat.1014341.s003.tif]

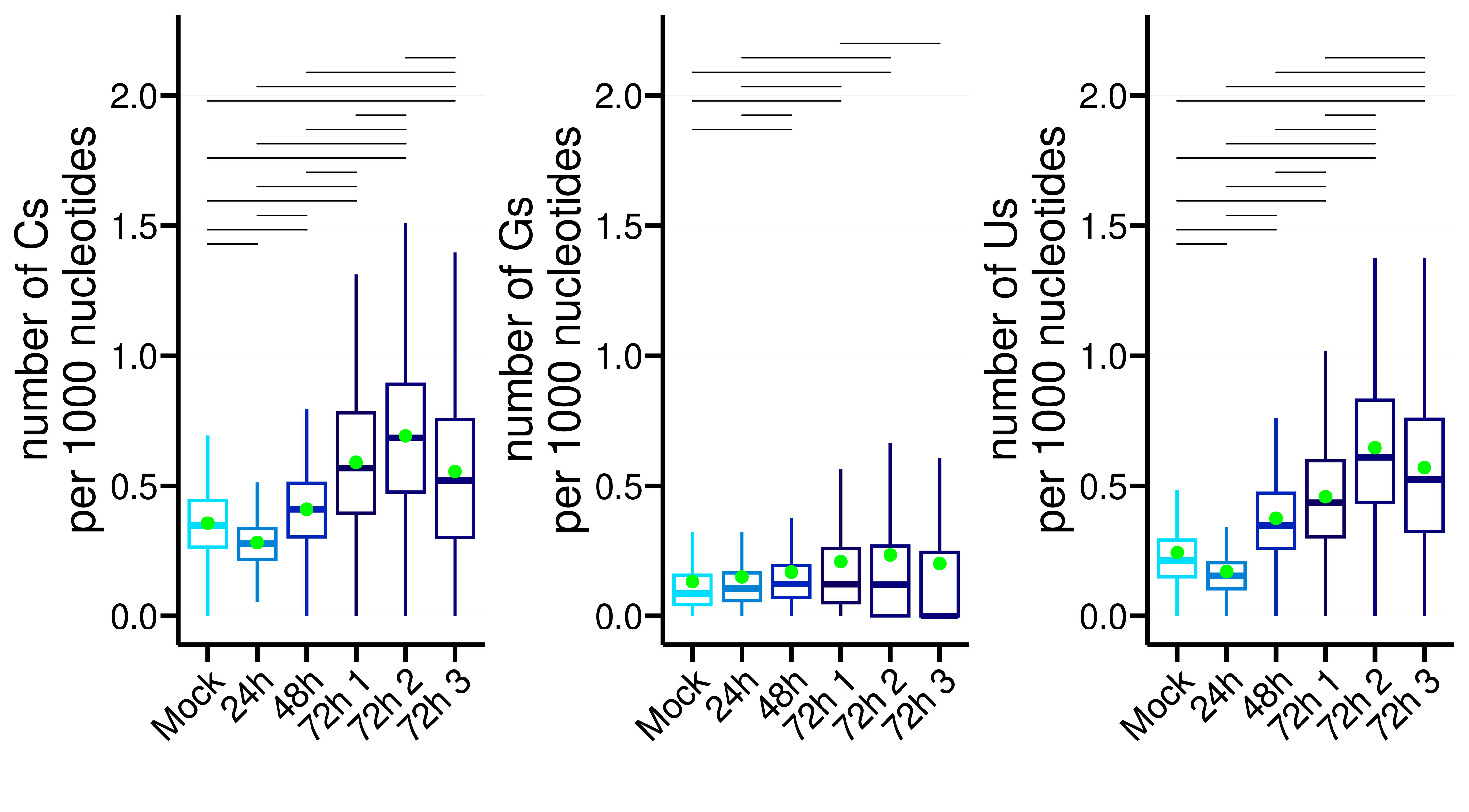

Supplement: S4 Fig — Frequency of non-adenosine residues per 1000 nucleotides in human mRNAs across different timepoints. A per mRNAs coverage filter of ≥30 was applied, only transcripts that fulfill this criterion across all samples are included. Boxplots whiskers depict 1.5 * IQR, the mean is shown by the green dot. Kruskal-Wallis rank sum test was applied on all groups for every of the three nucleotides, followed by a two-sided Dunn’s test with Bonferroni correction. Significant adjusted p-values (≤0.05) are marked above the boxplots, complete Z-scores and adjusted p-values can be found in S6 Table. (TIF) [file ppat.1014341.s004.tif]

Raw Images for blots in Figure S1

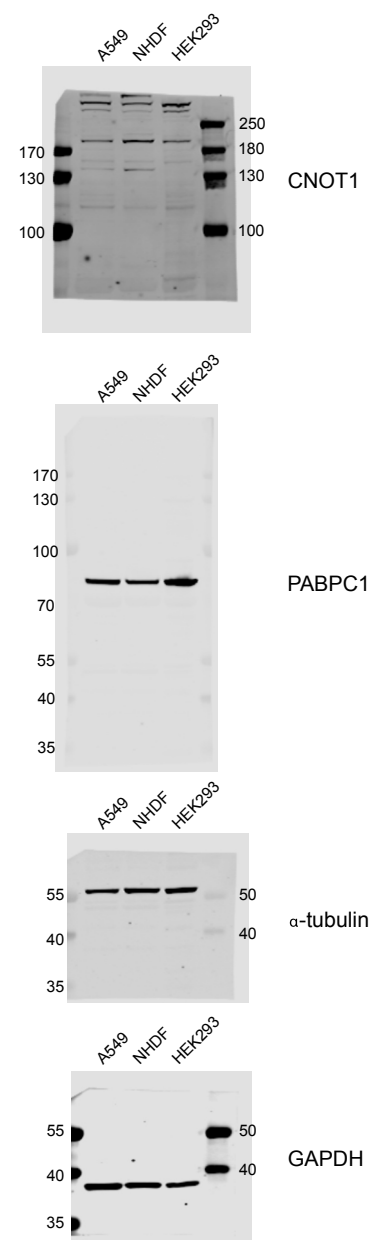

Supplement: S2 File — (PDF) [file ppat.1014341.s012.pdf]
