# Supplementary material for: Extended poly(A) tails are a shared feature of herpesvirus mRNAs
Source: PLoS Pathog. 2026 Jun 16;22(6):e1014341. doi: 10.1371/journal.ppat.1014341 (PMC13271517; doi:10.1371/journal.ppat.1014341)
Supplement: S1 File — (PDF) [file ppat.1014341.s011.pdf]

Supplementary statistics

This supplementary file contains additional statistical notes and tables associated with the manuscript yet. All statistics were calculated using R. For package versions, see the methods section in the manuscript. All scripts can be found at https://github.com/DepledgeLab/poly-A-tails.

Comparison of human vs. viral poly(A) tail lengths during infection in Figs. 1b-h and 3a.

Datasets	No. tested reads human	Mean human	Median human	Mode human	SD human	No. tested reads viral	Mean viral	Median viral	Mode viral	SD viral	W/U	P	CI	A
1b) HCMV	115,574	108.79	102.74	94	55.51	155,663	117.04	116.38	122	51.52	8,002,611,690	< 2.2e-16	-10.80 -9.96	0.44 (negligible)
1c) VZV	208,333	123.7	115.6	80	64.76	124,896	160.4	155.5	150	56.51	8,139,381,741	< 2.2e-16	-40.28 -39.47	0.31 (medium)
1d) HSV-1	276,460	96.34	80.00	42	63.10	443,542	138.2	137.6	142	52.45	3.4363e+10	< 2.2e-16	-49.57 -49.03	0.28 (medium)
1e) HSV-2	21,268	78.81	60.34	51	63.03	367,559	95.35	91.93	96	45.29	2,675,522,714	< 2.2e-16	-25.46 -24.27	0.34 (small)
1f) KSHV	301,721	99.40	90.87	52	55.25	30,280	116.78	108.04	104	66.98	3,885,452,861	< 2.2e-16	-15.34 -14.00	0.43 (small)
1g) EBV	24,063	98.02	86.85	57	59.49	4,356	106.40	100.25	102	62.51	47,970,779	< 2.2e-16	-10.17 -6.50	0.46 (negligible)
1h) HVS	40,620	62.19	51.30	50	49.38	386,374	132.39	126.72	122	67.36	2,791,223,025	< 2.2e-16	-70.19 -69.05	0.18 (large)
3a) SARS-CoV-2	8,244	159.17	146.35	170	91.05	31,226	80.82	63.42	54	54.49	201,631,279	< 2.2e-16	69.90 73.36	0.78 (large)

Table 1. Descriptive and test-statistic values are listed. SD = Standard deviation. W/U = W/U statistic of two-sided Wilcoxon rank sum test (Mann-Whitney U test). CI = non-parametric Confidence Interval of the location shift based on normal. A = Vargha and Delaney's A as effect size measure.

Comparison of human poly(A) tail lengths in different cell lines (Mock) in Fig. 1a.

**General metrics**				
AIC	BIC	logLik	-2*log(L)	df.resid
13,383,008	13,383,068	-6,691,499	13,382,998	1,249,027
**Fixed effects**				
	Estimate	Std. Error	T value	Pr(>|z|)
(Intercept)	4.8124	0.0018	2,602.41	<2e-16
RunMeWo	0.0397	0.0016	24.06	<2e-16
RunNHDF	-0.1317	0.0014	-93.16	<2e-16
**Random effects**				
	Variance	Std.Dev.	No. groups	
Transcript (Intercept)	0.0280	0.1672	30,109	
Residual	0.3168	0.5628		
**Estimated marginal means**				
Run	Response	SE	CI (95%)	
A549	123.0	0.228	122.6	123.5
MeWo	128.0	0.230	127.6	128.5
NHDF	107.8	0.172	107.5	108.2
Pairwise comparison				
contrast	Ratio	SE	Z ratio	p
A549 / MeWo	0.9611	0.0016	-24.055	<0.0001
A549 / NHDF	1.1408	0.0016	93.160	<0.0001
MeWo / NHDF	1.1869	0.0016	123.854	<0.0001

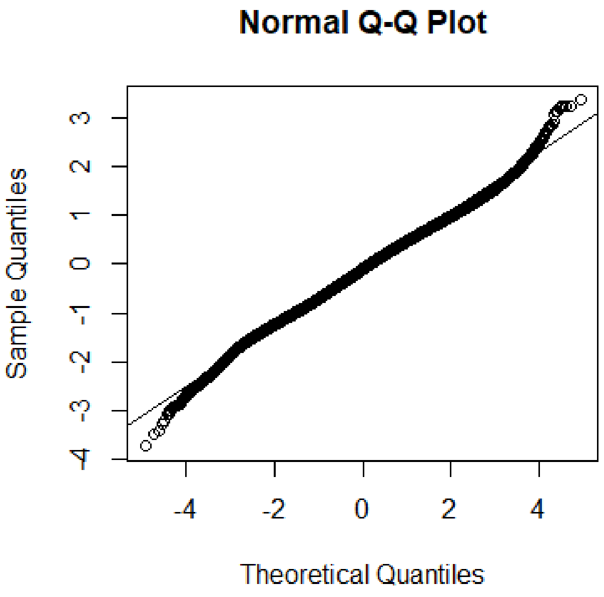

Table 2 (left). Metrics of the Generalised linear mixed model (GLMM). (*PolyA_Length ~ Run + (1 | Transcript*); Gamma family with log linkage function. AIC = Akaike Information Criterion. BIC = Bayesian Information Criterion. df = degrees of freedom. SE = Standard Error. CI = Confidence Interval. Estimated marginal means and pairwise comparison on response scale. Pairwise comparison p values corrected with Holm-Bonferroni.

Figure 1 (right). QQ-Plot of the GLMM models Pearson residuals plotted against theoretical normal quantiles. The QQ-plot of the same GLMM model with an identity linkage function showed a much higher deviation (not shown).

Comparison of human poly(A) tail lengths across timepoints in Fig. 3b.

**General metrics**				
AIC	BIC	logLik	-2*log(L)	df.resid
8,971,926	8,971,996	-4,485,957	8,971,914	849,186
**Fixed effects**				
	Estimate	Std. Error	T value	Pr(>|z|)
(Intercept)	4.783.802	0.0019	2,455.99	<2e-16
Run12	-0.0721	0.0016	-45.11	<2e-16
Run24	-0.1667	0.0018	-90.19	<2e-16
Run48	-0.3393	0.0020	-169.90	<2e-16
**Random effects**				
	Variance	Std.Dev.	No. groups	
Transcript (Intercept)	0.0273	0.1652	25650	
Residual	0.3106	0.5573		
**Estimated marginal means**				
Run	Response	SE	CI (95%)	
h0	119.56	0.233	119.10	120.02
h12	111.24	0.214	110.82	111.66
h24	101.20	0.216	100.78	101.62
h48	85.16	0.195	84.78	85.54
Pairwise comparison				
contrast	Ratio	SE	Z ratio	p
h0 / h12	1.075	0.0017	45.112	<0.0001
h0 / h24	1.181	0.0022	90.194	<0.0001
h0 / h48	1.404	0.0028	169.900	<0.0001
h12 / h24	1.099	0.0020	51.378	<0.0001
h12 / h48	1.306	0.0026	134.253	<0.0001
h24 / h48	1.188	0.0026	78.713	<0.0001

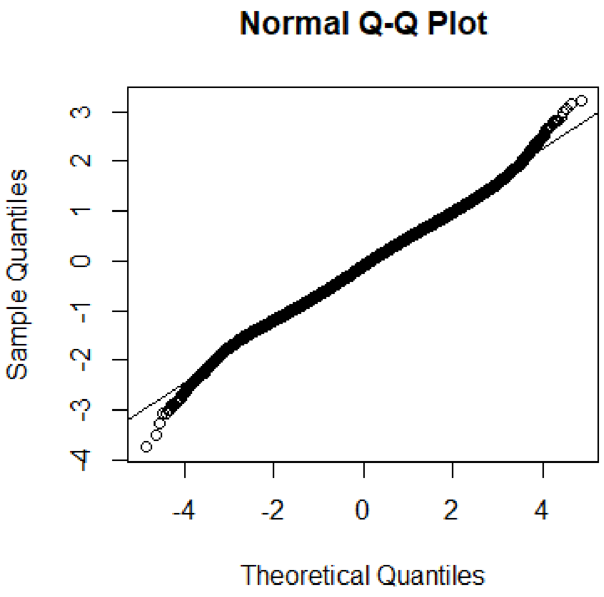

Table 3 (left). Metrics of the Generalised linear mixed model (GLMM). (*PolyA_Length ~ Run + (1 | Transcript*); Gamma family with log linkage function. AIC = Akaike Information Criterion. BIC = Bayesian Information Criterion. df = degrees of freedom. SE = Standard Error. CI = Confidence Interval. Estimated marginal means and pairwise comparison on response scale. Pairwise comparison p values corrected with Holm-Bonferroni.

Figure 2 (right). QQ-Plot of the GLMM models Pearson residuals plotted against theoretical normal quantiles. The QQ-plot of the same GLMM model with an identity linkage function showed a much higher deviation (not shown).

Comparison of human poly(A) tail lengths across timepoints in Fig. 3c.

**General metrics**				
AIC	BIC	logLik	-2*log(L)	df.resid
10,177,538	10,177,597	-5,088,764	10,177,528	958,155
**Fixed effects**				
	Estimate	Std. Error	T value	Pr(>|z|)
(Intercept)	4,633,910	0.0016	2,812,788	<2e-16
Run12	-0.0022	0.0016	-1.394	0.163
Run24	-0.0154	0.0019	-8.273	<2e-16
**Random effects**				
	Variance	Std.Dev.	No. groups	
Transcript (Intercept)	0.0269	0.1639	28,519	
Residual	0.3364	0.5800		
**Estimated marginal means**				
Run	Response	SE	CI (95%)	
h0	102.9	0.170	102.6	103.2
h12	102.7	0.204	102.3	103.1
h24	101.3	0.226	100.9	101.8
Pairwise comparison				
contrast	Ratio	SE	Z ratio	p
h0 / h12	1.002	0.0016	1.394	0.1634
h0 / h24	1.015	0.0019	8.273	<0.0001
h12 / h24	1.013	0.0022	6.109	<0.0001

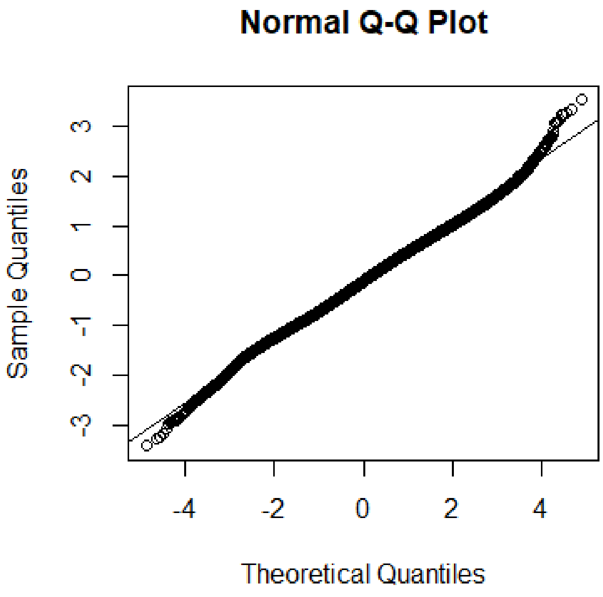

Table 4 (left). Metrics of the Generalised linear mixed model (GLMM). (*PolyA_Length ~ Run + (1 | Transcript*); Gamma family with log linkage function. AIC = Akaike Information Criterion. BIC = Bayesian Information Criterion. df = degrees of freedom. SE = Standard Error. CI = Confidence Interval. Estimated marginal means and pairwise comparison on response scale. Pairwise comparison p values corrected with Holm-Bonferroni.

Figure 3 (right). QQ-Plot of the GLMM models Pearson residuals plotted against theoretical normal quantiles. The QQ-plot of the same GLMM model with an identity linkage function showed a much higher deviation (not shown).

Comparison of human poly(A) tail lengths of individual transcripts in Fig. 2.

Two-sided Wilcoxon rank sum tests (Mann-Whitney U tests) were performed for every transcript with a depth >= 50 in both conditions. P-values were corrected with Holm-Bonferroni. Effect sizes were calculated via wilcoxons r (column effsize). Test statistics and per transcript poly(A) tail length median (changes) are shown in Table S2.
