# Supplementary material for: Extended poly(A) tails are a shared feature of herpesvirus mRNAs
Source: PLoS Pathog. 2026 Jun 16;22(6):e1014341. doi: 10.1371/journal.ppat.1014341 (PMC13271517; doi:10.1371/journal.ppat.1014341)
Supplement: Attachment — Submitted filename: Response to reviewer comments.pdf [file ppat.1014341.s013.pdf]

RESPONSE TO REVIEWERS

We thank the reviewers for their thoughtful comments on our manuscript and the opportunity to highlight our novel and mechanistic insights in a revised version. While broadly supportive, the reviewers noted concerns on the novelty, mechanistic insights and statistical assessment of our presented work.

To our knowledge, this is the first study to apply the same methodology consistently and systematically across nearly the full diversity of herpesviruses, enabling direct, like-for-like comparisons of poly(A) tail dynamics and mixed tailing and revealing a novel shared feature of herpesvirus transcripts. Importantly, while herpesviruses employ markedly different strategies to subvert host cell gene expression machinery, we here discover a convergent effect on poly(A) tail lengths, suggestive of a common underlying principle of host–virus interaction. This cross-family conservation is not predictable from existing literature and highlights a unifying layer of post-transcriptional regulation.

Our analyses also address the mechanism of extended herpesvirus mRNA poly(A) tails. Comparisons with adenovirus and other viruses demonstrate that an acute burst of RNAPII-driven viral transcription cannot account for extended tails, while our analysis of mixed tailing, now including analysis in cells with disrupted deadenylase activity, shows that this cannot broadly explain tail extension or resistance to deadenylation. We have now also incorporated robust statistical assessments (detailed below) that provide strong support for our conclusions.

Our resubmitted manuscript includes additional figures (Fig. 2, S1, S2, and revised versions of Figures 7 and 8, formerly 6 and 7), analyses and text revisions (indicated in red font).

Given the growing interest in RNA tailing, modification, and decay as key regulators of gene expression, we consider this work to be both timely and foundational. It provides a framework and dataset that will be of broad relevance to virologists studying host–pathogen interactions as well as to RNA biologists investigating mechanisms of RNA stability and processing in broad contexts, including RNA therapeutics.

Our point-by-point response (red text) to the reviewer's comments follows:

Reviewer #1:

1. the authors mention in the introduction that PABPC1 expression varies by cell type and this could be a major determinant of polyA tail length/stability: since they are using a variety of cell lines, it would be interesting to have a comparison of PABCP1 expression across these cell types and see if they can find a "pattern". It would be important to test the same herpesviral infection (of their choosing) in a different cell line and see if the longer polyA tails match across cell lines (overall longer polyA tails + specific viral transcripts having the same polyA tail length across multiple cell line).

We agree, we are very interested in how PABPC availability could contribute to herpes tail length control and dictate translational responsiveness to poly(A)-tail length. This cannot be reliably assessed at the transcript level within our data as PABPC1 is regulated translationally through an autoregulatory circuit (PMID: 16356927) and requires analysis of protein abundance. As this work was the result of an extensive international collaboration effort, we could not test all cell types used under the same conditions, however we have now assessed and compared both PABPC1 and major deadenylase complex CCR4-NOT1 scaffold CNOT1 protein levels in cell lines currently available in one contributing laboratory (A549, NHDF, HEK293) and now include these as Supplementary Figure 1, described on lines 270-273. We find that levels of both vary among the cell lines used, with primary fibroblast NHDF cells

showing higher CNOT1 levels and lower PABPC1 levels, while HEK293 cells showed relatively elevated PABPC1 abundance. Interestingly EBV tail lengths in HEK293 cells (modal length: 102nt) were among the shortest of the long herpes tails (150nt – 96 nt) consistent with elevated PABPC levels in those cells potentially contributing to enhanced deadenylation.

Unfortunately, the discontinuation of the Nanopore RNA0002 RNA sequencing kits and flowcells does not permit a repeat analysis of the same virus in a different cell line with identical sequencing conditions to our existing datasets. However, NHDF cells were used for infections with both HSV-1 and HCMV, which displayed different modal viral tail lengths (142nt and 122nt respectively), and effects on host mRNA tails, supporting an active role by specific herpes viruses to influence poly(A)-length dynamics in infected cells.

Whether the availability of PABPC1 and CCR4-NOT components in the cell types susceptible to each herpes virus or following viral manipulation of these cellular proteins results in differing outcomes of long poly(A) tails on viral (and cellular) mRNA translation and overall viral gene expression will be a subject of our follow-on investigations.

2. there are no statistical tests performed in any of the figures to compare host vs. viral polyA tail length. All their conclusions are drawn from violin plots being wider or median length being longer – without any test.

- “Notably, the shapes of the distributions also differed e.g. for HSV-2 the density of poly(A) tail lengths is more widely distributed, resulting in a narrower plot.” This is very vague, is there a way to quantify this distribution?

- line 239: “a slightly larger proportion of mRNAs in A549 cells have poly(A) tail lengths of ~52 nt when compared to NHDFs.” Is this significant? Is there a statistical test that can support this claim? If not, is it relevant? Same comment for line 290, 333…
- once again, it is hard to interpret the difference between Fig 1a A549 uninfected with an average polyA tail at 52, while when infected, it climbs up to 170 in Fig 2a: are we looking at an overall shift in polyA tail length, or a select portion of transcripts with significantly longer polyA tails? And just describing the “shape’ of the distribution is not quantitative enough.

We appreciate the reviewer highlighting the need to present statistical support when comparing poly(A) tail length distributions. To address this, we have incorporated comprehensive statistical analyses throughout the manuscript and added a new figure (Fig. 2) to further clarify how transcript-level changes contribute to population-level effects.

Specifically, we have applied appropriate statistical tests to compare poly(A) tail length distributions between conditions (including Mann–Whitney U tests and generalized linear mixed models where appropriate), quantified distribution characteristics using descriptive statistics (e.g., median and variance), and reported effect sizes (Vargha and Delaney’s A) alongside p-values and confidence intervals (see Supplementary Statistics). These analyses confirm that the observed differences between host and viral poly(A) tail length distributions are statistically significant and not solely based on visual interpretation of violin plots.

To address the important question of whether infection leads to a global shift in host poly(A) tail length or changes in specific subsets of transcripts, we introduced a new analysis (Fig. 2). Here, we restricted the analysis to transcript isoforms with sufficient coverage (>50 reads in both conditions) to support statistical analysis and compared median poly(A) tail lengths per transcript. This analysis reveals that, for VZV and HCMV infections, median poly(A) tail lengths generally exhibit a statistically significant increase upon infection with distinct subsets of transcripts showing increases in poly(A) tail length of ≥27 nt. By contrast, HSV-1 infections show that the majority (~88%) of the cellular mRNAs examined did not show statistically

significant changes in median poly(A) tail lengths. A limitation of this analysis is that the sequencing depths achievable with the methodology used here restricts our analysis to a subset of ~300-700 abundant cellular mRNAs). This demonstrates that the increase in average poly(A) tail length observed at the population level is primarily driven by a subset of transcripts rather than a uniform global shift. Functional enrichment analyses of mRNAs showing statistically significant changes in median poly(A) tail length did not identify significant enrichment for specific pathways for any of our datasets.

3. line 295-302: the authors make a point of explaining that the "age" of the transcript is "unlikely" to explain the longer polyA tails but they don't have data supporting this claim: either remove, include data (synchronize cells, pulse chase a transcriptional inhibitor…), or move to the discussion section.

We agree this possibility is better explored in the discussion alongside alternatives. We removed this section in the results section and have edited the language describing our adenovirus data to highlight that high multiplicity infections represent synchronized viral gene expression (lines 345-349). We thank the reviewer for prompting us to make this improvement.

4. Fig 3a: the width of the violin plots is unclear: in the leftmost plot, this is a comparison of all the viral mRNAs (so presumably many different mRNAs distributed over many different polyA lengths) then the next plots are just the distribution of polyA tail length of individual ncRNA. Yet, the width of the plot is the same. This is raising a lot of questions: if the authors capture multiple polyA tail lengths for each transcript (which would make sense), then where is this diversity plotted in the aggregated violin plot of "all mRNA" (here and in previous figures)? Or are the authors plotting just the average (median/modal) polyA tail length per mRNA in these violin plots? In which case, is it really correct to directly compare to the distribution of polyA tails of one single transcript?

All violin plots are based on poly(A) tail length measurements from individual RNA molecules. The "viral mRNAs" distribution represents pooled molecule-level measurements across all viral mRNAs, whereas the other violins show molecule-level distributions for individual transcripts. Thus, in all cases, the full diversity of poly(A) tail lengths is represented, rather than summary statistics per transcript. Importantly, violin width reflects the density of observations and is independent of the total number of molecules. Because all distributions are shown at the same (molecule-level) resolution, direct comparison between pooled mRNAs and individual transcripts is appropriate. We have also added this clarification to the relevant figure legends.

5. the authors conclude that because the couple HCMV nuclear retained ncRNAs that they selected also had longer polyA tails despite being nuclear that "herpes RNA tail lengths are not singularly defined by nuclear retention or translation status". This is a generalization and would need a lot more thorough comparison to conclude this. What about mRNA that are exclusively cytoplasmic or nuclear retained? What about the differential regulation and transcription rates of ncRNA? What about stability of ncRNA vs. mRNA independently of polyA length?

We agree. Our conclusion is not that poly(A) tail length is generally independent of nuclear retention or translation status, but rather that it cannot be only or strictly determined by these factors for herpesviral RNAs. The nuclear-retained HCMV ncRNAs with long poly(A) tails we identify present a direct counterexample to such a deterministic model. This nuance was lacking in our wording, and we have now provided a revised and clarified concluding statement to this section (line 382-385).

6. line 341: the authors state “three transcripts […] showed evidence of median poly(A) tail lengths increasing by ≥ 27 nt between at least two timepoints” – this is too vague. When looking at the graph, it looks like there is a lot of variability (US12 polyA tail is longer at 6h p.i. then shorter at 12h p.i.; it’s the reverse for ICP4…). The authors should refrain for generalizing their data.

We have addressed this by specifically delineating the changes for each of these three mRNAs over the three time points examined (lines 399-403).

7. line 353: the authors state “RL2 (ICP0) mRNA has previously been reported to display nuclear retention (53). This partial segregation from the cellular cytoplasmic deadenylation machinery may thus explain this sustained increase in poly(A) length.” But the authors do not show any additional data to support their speculation. Either remove, show data (relieve ICP0 nuclear retention and check its polyA tail) or move to the discussion section.

We have now moved this speculative comment to the discussion.

8. fig 6: the authors conclude that broadly, longer polyA tail length in herpesviruses does not correlate with higher rates of mixed tails: it would be interesting to check at the viral level. Are the viral transcripts with the longest polyA tail more prone to mix tailing? Or is it also randomly distributed across all viral transcripts?

The reviewer raises an interesting point regarding whether, within individual herpesviruses, transcripts with longer poly(A) tails exhibit higher rates of mixed tailing. However, interpreting such a relationship is non-trivial because poly(A) tail length and nonA content are not independent variables. First, different models of nonA incorporation lead to opposing expectations. If nonA incorporation occurs stochastically along the tail, longer poly(A) tails will inherently contain more nonA residues simply due to their length, requiring normalization by tail length. By contrast, if nonAs are incorporated at defined positions (e.g., near the 3′ end), absolute nonA counts become more relevant, and length normalization would introduce bias. Second, and more importantly, poly(A) tails are dynamic, and the observed tail length does not reflect the original length at synthesis. Because transcripts undergo deadenylation over time, shorter tails may represent older molecules that have already lost terminal nonA residues. Conversely, some longer tails may correspond to younger transcripts that have not yet undergone deadenylation. This creates an inherent bias whereby longer tails may appear enriched for nonA residues even in the absence of a causal relationship. As a result, correlations between observed poly(A) tail length and nonA frequency should not be interpreted as reflecting functional relationships and it could be misleading to present such data.

9. it is unclear if the authors have taken into account the relative expression level of their herpesviral transcripts across time points.

We have now performed additional analyses of the HCMV and HSV-1 time-course data that examines the relationship between relative abundance (TPM) and median poly(A) tail length. We observed no significant correlation for HCMV. In contrast, HSV-1 showed a moderate negative correlation, most pronounced at early infection (3 hpi). Further analysis indicates that this effect is largely driven by highly abundant immediate-early (IE) transcripts, which exhibit comparatively less extreme poly(A) tail lengths at this time point. These results are now presented in a new supplementary figure (Fig. S2, lines 404-408) and provide a rationale for our subsequent analysis of poly(A) tail length dynamics across temporal gene classes (Figs 5 and 6). We have clarified this in the revised manuscript.

Reviewer #2: Specific recommendations:

- The authors claim that longer mRNA tails advantage herpesvirus gene expression (abstract), but no evidence is provided in this study. This claim should be removed, or additional functional data provided.

We agree with the reviewer that though longer poly(A)-tails are canonically associated with increased mRNA stability, translation and overall expression, we have not specifically demonstrated this is also true in the infection contexts studied here. We have therefore rephrased this statement in our abstract to make clear this is just a potential outcome of the extended poly(A)-tails in herpesvirus mRNAs we have discovered.

- The authors claim that longer mRNA tails do not correlate with nuclear localisation (abstract). This is based on the analysis of a few nuclear localised transcripts, and is too general for the evidence provided. The authors should weaken this claim, or provide additional data on tail length, for example by performing nuclear / cytoplasmic fractionation.

We agree with the reviewer that we do not have sufficient evidence to make claims for such links for herpesviral messenger RNAs, though intend to investigate this in the future. We hope our reworded abstract now more clearly states that we find no correlation between the localisation and poly(A)-tail status of the limited number of herpesvirus non-coding RNAs.

- The authors claim elongated polyA tails are a conserved feature of herpesviruses. The claim is a bit too strong. Current evidence points to multiple mechanisms leading to tail elongation, and it is not clear whether each of these is conserved in a true sense ... maybe shift to "elongated polyA tails are observed across representatives of the main herpesvirus sub-families". In the discussion the authors use "general feature" which is a better descriptor than conserved in this context.

We agree with the reviewer's point and have revised the text to reflect this on line 93.

- Replication. It is quite unclear how many replicates the authors are using. For example, Figure 4 and Figure 5 show transcript specific modulation of polyA tail length. These data can be obtained from pooled single molecule measurements providing a robust measurement with error, however, are the data considering replication of individual time points? The observed differences are very slight: are they reproducible across independent replicates?

Datasets for HCMV infection of NHDFs at 72 hpi (three biological replicates) and for HCMV-infected NHDFs following CNOT1 silencing (two biological replicates) show highly similar poly(A) tail length distributions across replicates. These observations provide confidence that poly(A) tail length measurements are reproducible and that the distributions are largely consistent between biological replicates. Except for the 72hpi HCMV datasets, all other samples analysed in this study are single biological replicates. Replicate numbers are now made clear in our legends and methods section. Unfortunately, the discontinuation of the RNA002 chemistry and R9.4 flowcells in 2025 prevents the generation of additional replicates.

- Transcript specific features. In this study, polyA tail lengths of the viral transcripts do not appear to change substantially at different infection stages (fig 4 and 5). Given that host changes in the transcriptome are well described in the literature using less powerful approaches, it seems like a missed opportunity by the authors to not investigate full length isoform changes and heterogeneity in the host cell transcriptome across the different viruses tested. Could the authors comment on why this was not of interest - the team clearly has the knowledge and capabilities to address this, and the analysis might reveal mechanistic details of increase polyA tail lengths.

We thank the reviewer for this suggestion that led us to further analyse our datasets and add additional insights to our manuscript. We initially did not pursue this due to the limitation of host transcriptome sequencing depth associated with R9.4.1 flowcells and the ONT mk1.b MinION, which generally yielded 750k-1.5m reads, of which a substantial fraction derived from viral RNAs. However, we have now performed analysis of poly(A) tail length dynamics on the subsets of cellular transcripts for which sufficient read depth is available in infected and uninfected samples and include this as a new Figure 2, discussed on lines 299-320. This analysis indicates that infection with VZV and HCMV is associated with an overall increase in median poly(A) tail length, driven in part by specific subsets of transcripts that display elongation of ≥27 nt. By contrast, during HSV-1 infection, most cellular mRNAs examined (~88%) do not exhibit significant changes in their median poly(A) tail lengths. Together, these findings suggest that the observed increase in average poly(A) tail length at the population level is not due to a uniform shift across all transcripts but rather reflects changes occurring within defined subsets. Functional enrichment analysis of transcripts showing significant tail length increases did not reveal enrichment for any specific biological pathways across the datasets. It is important to note that this analysis is limited by sequencing depth, which restricts assessment to approximately 300–700 relatively abundant cellular transcripts.

We aim in the future to extend these analyses to a full investigation of poly(A) tail dynamics across the host transcriptome, including their relationships with splice isoforms, alternative polyadenylation, and RNA modifications. Achieving this however will require more than a tenfold increase in sequencing depth, which we hope will be enabled by recent advances in ONT RNA sequencing chemistry, flow cells and instrumentation.

- Additional functional data. The inclusion of additional functional data would transform the manuscript from descriptive to mechanistic. Are polyA tail longer / shorter in key cellular compartments (E.g. by isolation and sequencing of RNA in polysomes to support involvement in translation)? Alternatively, how do polyA tail length relate to transcript stabilities or life times e.g. using 4SU experiments (difficult) or RNA velocity measurements (theoretically possible with dRNA). Are PABPs enriched on viral transcripts? Extensive additional functional assays may not be needed if host transcriptome changes (see point above) are sufficiently novel.

We believe this foundational work, revealing for the first time extended poly(A) tails as a shared feature herpes virus mRNAs, is an important first step which lays the groundwork for future investigation of potential shared or divergent causes of extended tails, and consequences for host and viral gene expression. Integrating poly(A) tail length and RNA stability and translatomic data during viral infections requires extensive assay development but is part of our future plans. Nevertheless, we believe our analyses here <u>have</u> provided mechanistic insights into the regulation of poly(A)-tail length during herpesviral infection. By comparison with other viruses, specifically the nuclear replicating DNA adenovirus, we demonstrate that a surge of new viral RNAPII-derived transcripts is insufficient to produce a viral transcriptome with extended tails (Fig 4). By segmenting viral transcripts by temporal class, and distinct timepoints this conclusion is supported and excludes the possibility of promoter-specific determination of poly(A)-tail length (Figs 5, 6). By analysing the incidence of mixed tailing, we show this cannot provide a broad explanation for herpesvirus mRNA poly(A) tail extension or resistance to deadenylation (Figs 7, 8). While many questions remain, we believe these findings provide a meaningful starting point and merit dissemination to the research community and PLoS Pathogens readership.

Minor Points:

Reviewer #1:

1. given that some of the data was collected from publicly available datasets and some were newly generated, it would be important to confirm that the RNA was prepared similarly (using the same dynabead for RNA isolation? All dataset started from 25ug of RNA?)

We have now amended the text description in the methods to make it clear that all datasets used in this study were generated by our lab and that this study comprises a mixture of previously published (i.e. publicly available) and new datasets that, in all cases, were generated using the same amounts of starting material and dynabeads.

2. Fig 1 should also show the length of the polyA tails on human transcript with and without infection (not just Table S1).

We have now made clear the modal lengths on human mRNAs with and without infection are both indicated in Figure 1 and appropriately referenced in the results text.

3. in the method section (and the start of the result section), the authors describe using A549 cells for CoV2 infection but say that the CoV2 data was collected from publicly available datasets and do not describe CoV2 infections. At the start of the result section, they also don't mention the SLK cells used for KSHV infection, the HEK293 cells used for EBV infection, the ARPE-19 cells used for HSV2. Altogether, this comes out as confusing as to which infection they performed, which cells they extracted RNA from etc. Please streamline. And include all cell lines in Fig1A for accurate comparison.

We thank the reviewer for their feedback regarding the lack of clarity around our data sources and generation. We have rephrased our methods regarding cell lines handled in the generation of new sequencing data presented here. We regret we do not have access to additional RNA002 chemistry and R9.4 flowcells to perform additional sequencing on uninfected cells from every infection model used here as they are now superseded by the RNA004 chemistry and RNA flowcells. However, given the similarity of the human transcriptome poly(A)-tail distribution observed in the three assessed uninfected cells (modal lengths 52, 54, 48 nts from A549, MeWO and NHDF respectively) and to published human cell line datasets using this and other poly(A)-tail measuring approaches, we do not believe this would offer significant additional insights.

4. line 280: typo in sgRNA – it should be sgmRNA

We have fixed this typo.

5. line 308-309: rephrase: it is unclear what a "polyA tail of differing length" means.
We have reworded this phrase for clarity.

6. for consistency, fig 3A/3B should show the "modal" tail length on the graph
This has been amended.
