# Supplementary material for: Extended poly(A) tails are a shared feature of herpesvirus mRNAs
Source: PLoS Pathog. 2026 Jun 16;22(6):e1014341. doi: 10.1371/journal.ppat.1014341 (PMC13271517; doi:10.1371/journal.ppat.1014341)
Supplement: Attachment — Submitted filename: Response_to_reviewer_comments_auresp_2.pdf [file ppat.1014341.s014.pdf]

RESPONSE

We thank the academic editor and reviewer for their consideration of our revised manuscript and are happy to address the remaining minor concerns.

Our point-by-point responses (red text) follow:

Reviewer #1:

1. PABPC expression across cell types: The authors have now included PABPC expression analyses in three cell lines and explain that additional cell lines could not be assessed. However, in their response, they propose that shorter EBV poly(A) tail lengths could correlate with higher PABPC expression levels. This is again speculative, as no experimental data directly support such link. In the manuscript, the authors state that this "could explain their slightly lower overall modal mRNA poly(A) tail length," but this interpretation would require functional validation (for example, PABPC or CCR4 depletion experiments). Moreover, given that only three cell lines were analyzed, the evidence is insufficient to establish a meaningful correlation between PABPC expression and poly(A) tail length at this stage.

Published siRNA and degron depletion studies have directly shown that reduced PABPC levels results in global extended median poly(A)-tail length in mammalian cells (PMID: 29932901, 34213414). Likewise, the role of CCR4-NOT as the predominant cellular deadenylase was directly shown by knockdown studies where global poly(A) tail extension was observed (PMID: 29932901). Moreover, we previously demonstrated in NHDFs that knockdown of CCR4-NOT scaffold CNOT1 resulted in a stark upward shift in host mRNA poly(A) tail length using nanopore DRS (PMID: 37846490). We have now referenced these publications and tempered our language regarding Fig S1 at lines 285-287 as follows: "The abundance of these factors has been linked to global poly(A) tail lengths and thus may contribute to the small differences in modal mRNA poly(A) tail detected between cell lines."

2. Conclusions regarding cytoplasmic localization (lines 398–401): The authors state that "translation or cytoplasmic localisation appear not to be prerequisites for extended poly(A) tail lengths on herpesviral transcripts." I remain concerned that this conclusion is too broad relative to the data presented. Both Reviewer 2 and I raised this issue during the initial review, yet no additional experiments were included to directly support this claim (cell fractionation for example). As written, the statement reads as a general mechanistic conclusion that is not sufficiently substantiated by the current data.

We accept that while the localisation of several herpes noncoding and coding transcripts have been characterized by others, we have not recapitulated those results here and have removed our conclusion in the results section regarding localisation and translation, previously at lines 398-401. We have also removed reference to nuclear localised ncRNAs in the abstract (lines 38-39).

3. CNOT1 silencing experiments (Figure 7): It appears that the CNOT1 silencing experiments were performed only in duplicate. The authors should include a western blot demonstrating the efficiency of the knockdown. In addition, it is unclear whether the data presented in Figure 7 represent a single experiment or the average of biological replicates. The figure legend should clarify this point. The legend also does not clearly indicate which cell type was used for the silencing experiments, and the Methods section does not include how the knockdown was performed.

As described in the Methods and shown in Table S1, the CNOT1 datasets used here were previously published (PMID: 37846490) and that study included western blot confirmation that

the knockdown was efficient, in addition to the dramatic extension to host poly(A) tail length which was detected in these samples. Both replicates are shown separately in Figure 7a-b. The data shown in Figure 7c-d are labelled as HCMV (72h 1) siCNOT1 which represent one of the biological replicates with the other biological replicate shown in Figure S3a-b. The legends of Figure 7 and Fig S3 have been adjusted to explain that all experiments were performed in NHDFs.

Editor Comments

Authors should carefully temper the language throughout the manuscript to avoid overinterpretation of the findings.

We have now removed the statements in the results sections and abstract that our reviewers objected to.

In addition, validation of the knockdown presented in Figure 7 will be important for strengthening the conclusions.

Please see our response to point 3 above.

Journal Requirements:

1. Update Financial Disclosures on submission form

This has been addressed by email, as instructed

2. Provide the original uncropped and unadjusted images

These are now provided in Supplementary Information File 1
