# Supplementary material for: Extended poly(A) tails are a shared feature of herpesvirus mRNAs
Source: PLoS Pathog. 2026 Jun 16;22(6):e1014341. doi: 10.1371/journal.ppat.1014341 (PMC13271517; doi:10.1371/journal.ppat.1014341)
Supplement: Peer review history

# Peer review: Decision Letter 0

**DOI:** 10.1371/journal.ppat.1014341.r001

**Authors:** John Karijolich

4 Feb 2026

-->PPATHOGENS-D-26-00002

Extended poly(A) tails are a shared feature of herpesvirus mRNAs

PLOS Pathogens

Dear Dr. Depledge,

Thank you for submitting your manuscript to PLOS Pathogens. After careful consideration, we feel that it has merit but does not fully meet PLOS Pathogens's publication criteria as it currently stands. Therefore, we invite you to submit a revised version of the manuscript that addresses the points raised during the review process.

Please submit your revised manuscript by Apr 05 2026 11:59PM. If you will need more time than this to complete your revisions, please reply to this message or contact the journal office at plospathogens@plos.org. When you're ready to submit your revision, log on to https://www.editorialmanager.com/ppathogens/ and select the 'Submissions Needing Revision' folder to locate your manuscript file.

Please include the following items when submitting your revised manuscript:

* A letter that responds to each point raised by the editor and reviewer(s). You should upload this letter as a separate file labeled 'Response to Reviewers'. This file does not need to include responses to any formatting updates and technical items listed in the 'Journal Requirements' section below.

* A marked-up copy of your manuscript that highlights changes made to the original version. You should upload this as a separate file labeled 'Revised Manuscript with Track Changes'.

* An unmarked version of your revised paper without tracked changes. You should upload this as a separate file labeled 'Manuscript'.

If you would like to make changes to your financial disclosure, competing interests statement, or data availability statement, please make these updates within the submission form at the time of resubmission. Guidelines for resubmitting your figure files are available below the reviewer comments at the end of this letter.

We look forward to receiving your revised manuscript.

Kind regards,

John Karijolich, Ph.D.

Academic Editor

PLOS Pathogens

Blossom Damania

Section Editor

PLOS Pathogens

Sumita Bhaduri-McIntosh

Editor-in-Chief

PLOS Pathogens

orcid.org/0000-0003-2946-9497

Michael Malim

-->Editor-in-Chief

PLOS Pathogens

orcid.org/0000-0002-7699-2064

**Journal Requirements:**

If the reviewer comments include a recommendation to cite specific previously published works, please review and evaluate these publications to determine whether they are relevant and should be cited. There is no requirement to cite these works unless the editor has indicated otherwise.

1) Please ensure that the CRediT author contributions listed for every co-author are completed accurately and in full.

At this stage, the following Authors/Authors require contributions: Erik Fuhrmann, Sae Toda, Jonas Leins, Pierina Cetraro, Vedang Deshpande, Carina Jacobsen, Kai A Kropp, Mart M Lamars, Elene Loliashvili, Mostefa Saleban, Ruth Verstraten, Carolin Vogt, Wiyada Wongwiwat, Werner JD Ouwendijk, Abel Viejo-Borbolla, Rob E White, Angus C Wilson, Hannah M Burgess, and Daniel P Depledge. Please ensure that the full contributions of each author are acknowledged in the "Add/Edit/Remove Authors" section of our submission form.

The list of CRediT author contributions may be found here: https://journals.plos.org/plospathogens/s/authorship#loc-author-contributions

2) We ask that a manuscript source file is provided at Revision. Please upload your manuscript file as a .doc, .docx, .rtf or .tex. If you are providing a .tex file, please upload it under the item type u2018LaTeX Source Fileu2019 and leave your .pdf version as the item type u2018Manuscriptu2019.

3) Please provide an Author Summary. This should appear in your manuscript between the Abstract (if applicable) and the Introduction, and should be 150-200 words long. The aim should be to make your findings accessible to a wide audience that includes both scientists and non-scientists. Sample summaries can be found on our website under Submission Guidelines:

https://journals.plos.org/plospathogens/s/submission-guidelines#loc-parts-of-a-submission

4) Please upload all main figures as separate Figure files in .tif or .eps format. For more information about how to convert and format your figure files please see our guidelines:

https://journals.plos.org/plospathogens/s/figures

5) We have noticed that you have uploaded Supporting Information files, but you have not included a list of legends. Please add a full list of legends for your Supporting Information files after the references list.

6) In the online submission form, you indicated that your data will be submitted to a repository upon acceptance. We strongly recommend all authors deposit their data before acceptance, as the process can be lengthy and hold up publication timelines. Please note that, though access restrictions are acceptable now, your entire minimal dataset will need to be made freely accessible if your manuscript is accepted for publication. This policy applies to all data except where public deposition would breach compliance with the protocol approved by your research ethics board. If you are unable to adhere to our open data policy, please kindly revise your statement to explain your reasoning and we will seek the editor's input on an exemption.

**Reviewers' Comments:**

Reviewer's Responses to Questions

**Part I - Summary**

Please use this section to discuss strengths/weaknesses of study, novelty/significance, general execution and scholarship.

Reviewer #1: This article (PPATHOGENS-D-26-00002) by Furmann et. Al. explores polyA tail length during infection by various Herpesviruses (HCMV, HSV1 from previously published dataset and VZV, KSHV, EBV, HVS) as well as other viruses (Human adenovirus, SARS-CoV2 from previously published datasets and de novo MPVX) using nanopore sequencing. They show that polyA tails are longer on herpesviral transcripts than on human mRNA. They find non-adenosine residues in herpesviral tails, which could oppose deadenylation and explain their length. However, they say that this is not significant enough or correlated enough with polyA tail length to explain the widespread extended tails of herpesvirus mRNAs. They also discuss how there is no correlation between polyA tail and cytoplasmic vs. nuclear localization on ncRNAs and the heterogeneity of polyA tail lengths throughout infection. Finally, they conclude that the longer polyA tails of herpesviruses is not driven by a "mixed-tailing strategy” which is an interesting negative result but they do not offer any alternative.

This is an interesting story using nanopore technology to quickly and efficient address the complex question of polyA tail length. However, at this stage, I find this story to be highly descriptive, drawing conclusions on observations without robust statistical assessments which leads the authors to extrapolate based on their sequencing results. The lack of mechanistic insights limits the impact of this story.

Reviewer #2: In this manuscript, Fuhrmann et al. present an observational study on polyA tail lengths in host and viral RNAs, using diverse viruses and cell lines. They provide evidence of (i) virus induced changes in polyA tail length in host mRNA upon HCM, VZV and SARS-CoV-2 infection, (ii) that diverse herpesvirus mRNA have elongated tails compared to host mRNAs, and (iii) viral mRNAs have similar tail lengths to host mRNA in SARS-CoV-2, hADv41 and Mpox. Further investigation for HCMV identified long tails on non-coding transcripts and weak associations between tail length and time post infection and/or with mRNA class (IE, E vs L). Finally, the authors identify mixed tailing in a subset of viral and host mRNAs.

The strength of this study is its systematic application of dRNA-seq and analysis to a diverse set of pathogens and host cell line. It is clearly written and easy to understand. The discussion was excellent. Figures were of high quality, with the exception of figure 7, which was not so easy to read.

A weakness was overall novelty. (i) polyA tail length increases in virus and host has been already observed in different herpesvirus, and (ii) the new manuscript does not provide new mechanistic insights into these observations. In HCMV and HSV-2 polyA changes were linked to changed in host-RNA binding protein CPEB1 (PMID: 27775709). KSHV infection remodels host poly(A) (PMID: 19468299), with changes linked to expression of SOX. In HCMV, the same authors used dRNA-seq to link changes in polyA tail length to the CCR4-NOT deadenylase complex. In PMID: 32451488, HCMV transcripts were shown to contain mixed tails, which was attributed to TENT enzymes.

For publication in PLoS, I would expect new novel insights, particularly emerging from their application of dRNA-seq. That is, a major advantage of dRNA-seq over comparable methods e.g. tail-seq, is that polyA tail measurements can be linked directly to specific transcripts, along with their associated features (UTR lengths, modification, AS and AP site usage). Alternatively, new insight could be provided by additional functional data to exclude or support the mechanisms of tail length modulation beyond mixed tailing, which was already performed for HCMV.

**********

**Part II – Major Issues: Key Experiments Required for Acceptance**

Please use this section to detail the key new experiments or modifications of existing experiments that should be absolutely required to validate study conclusions.

Generally, there should be no more than 3 such required experiments or major modifications for a "Major Revision" recommendation. If more than 3 experiments are necessary to validate the study conclusions, then you are encouraged to recommend "Reject".

Reviewer #1: 1. the authors mention in the introduction that PABPC1 expression varies by cell type and this could be a major determinant of polyA tail length/stability: since they are using a variety of cell lines, it would be interesting to have a comparison of PABCP1 expression across these cell types and see if they can find a “pattern”. It would be important to test the same herpesviral infection (of their choosing) in a different cell line and see if the longer polyA tails match across cell lines (overall longer polyA tails + specific viral transcripts having the same polyA tail length across multiple cell line)

2. there are no statistical tests performed in any of the figures to compare host vs. viral polyA tail length. All their conclusions are drawn from violin plots being wider or median length being longer – without any test.

- “Notably, the shapes of the distributions also differed e.g. for HSV-2 the density of poly(A) tail lengths is more widely distributed, resulting in a narrower plot.” This is very vague, is there a way to quantify this distribution?

- line 239: “a slightly larger proportion of mRNAs in A549 cells have poly(A) tail lengths of ~52 nt when compared to NHDFs.” Is this significant? Is there a statistical test that can support this claim? If not, is it relevant? Same comment for line 290, 333…

- once again, it is hard to interpret the difference between Fig 1a A549 uninfected with an average polyA tail at 52, while when infected, it climbs up to 170 in Fig 2a: are we looking at an overall shift in polyA tail length, or a select portion of transcripts with significantly longer polyA tails? And just describing the “shape’ of the distribution is not quantitative enough.

3. line 295-302: the authors make a point of explaining that the “age” of the transcript is “unlikely” to explain the longer polyA tails but they don’t have data supporting this claim: either remove, include data (synchronize cells, pulse chase a transcriptional inhibitor…), or move to the discussion section.

4. Fig 3a: the width of the violin plots is unclear: in the leftmost plot, this is a comparison of all the viral mRNAs (so presumably many different mRNAs distributed over many different polyA lengths) then the next plots are just the distribution of polyA tail length of individual ncRNA. Yet, the width of the plot is the same. This is raising a lot of questions: if the authors capture multiple polyA tail lengths for each transcript (which would make sense), then where is this diversity plotted in the aggregated violin plot of “all mRNA” (here and in previous figures)? Or are the authors plotting just the average (median/modal) polyA tail length per mRNA in these violin plots? In which case, is it really correct to directly compare to the distribution of polyA tails of one single transcript?

5. the authors conclude that because the couple HCMV nuclear retained ncRNAs that they selected also had longer polyA tails despite being nuclear that “herpes RNA tail lengths are not singularly defined by nuclear retention or translation status”. This is a generalization and would need a lot more thorough comparison to conclude this. What about mRNA that are exclusively cytoplasmic or nuclear retained? What about the differential regulation and transcription rates of ncRNA? What about stability of ncRNA vs. mRNA independently of polyA length?

6. line 341: the authors state “three transcripts […] showed evidence of median poly(A) tail lengths increasing by ≥ 27 nt between at least two timepoints” – this is too vague. When looking at the graph, it looks like there is a lot of variability (US12 polyA tail is longer at 6h p.i. then shorter at 12h p.i.; it’s the reverse for ICP4…). The authors should refrain for generalizing their data.

7. line 353: the authors state “RL2 (ICP0) mRNA has previously been reported to display nuclear retention (53). This partial segregation from the cellular cytoplasmic deadenylation machinery may thus explain this sustained increase in poly(A) length.” But the authors do not show any additional data to support their speculation. Either remove, show data (relieve ICP0 nuclear retention and check its polyA tail) or move to the discussion section.

8. fig 6: the authors conclude that broadly, longer polyA tail length in herpesviruses does not correlate with higher rates of mixed tails: it would be interesting to check at the viral level. Are the viral transcripts with the longest polyA tail more prone to mix tailing? Or is it also randomly distributed across all viral transcripts?

9. it is unclear if the authors have taken into account the relative expression level of their herpesviral transcripts across time points.

Reviewer #2: Specific recommendations:

- The authors claim that longer mRNA tails advantage herpesvirus gene expression (abstract), but no evidence is provided in this study. This claim should be removed, or additional functional data provided.

- The authors claim that longer mRNA tails do not correlate with nuclease localisation (abstract). This is based on the analysis of a few nuclear localised transcripts, and is too general for the evidence provided. The authors should weaken this claim, or provide additional data on tail length, for example by performing nuclear / cytoplasmic fractionation.

- The authors claim elongated polyA tails are a conserved feature of herpesviruses. The claim is a bit too strong. Current evidence points to multiple mechanisms leading to tail elongation, and it is not clear whether each of these is conserved in a true sense ... maybe shift to "elongated polyA tails are observed across representatives of the main herpesvirus sub-families". In the discussion the authors use "general feature" which is a better descriptor than conserved in this context.

- Replication. It is quite unclear how many replicates the authors are using. For example, Figure 4 and Figure 5 show transcript specific modulation of polyA tail length. These data can be obtained from pooled single molecule measurements providing a robust measurement with error, however, are the data considering replication of individual time points? The observed differences are very slight : are they reproducible across independent replicates?

- Transcript specific features. In this study, polyA tail lengths of the viral transcripts do not appear to change substantially at different infection stages (fig 4 and 5). Given that host changes in the transcriptome are well described in the literature using less powerful approaches, it seems like a missed opportunity by the authors to not investigate full length isoform changes and heterogeneity in the host cell transcriptome across the different viruses tested. Could the authors comment on why this was not of interest - the team clearly has the knowledge and capabilities to address this, and the analysis might reveal mechanistic details of increase polyA tail lengths.

- Additional functional data. The inclusion of additional functional data would transform the manuscript from descriptive to mechanistic. Are polyA tail longer / shorter in key cellular compartments (E.g. by isolation and sequencing of RNA in polysomes to support involvement in translation)? Alternatively, how do polyA tail length relate to transcript stabilities or life times e.g. using 4SU experiments (difficult) or RNA velocity measurements (theoretically possible with dRNA). Are PABPs enriched on viral transcripts? Extensive additional functional assays may not be needed if host transcriptome changes (see point above) are sufficiently novel.

**********

**Part III – Minor Issues: Editorial and Data Presentation Modifications**

Please use this section for editorial suggestions as well as relatively minor modifications of existing data that would enhance clarity.

Reviewer #1: 1. given that some of the data was collected from publicly available datasets and some were newly generated, it would be important to confirm that the RNA was prepared similarly (using the same dynabead for RNA isolation? All dataset started from 25ug of RNA?)

2. Fig 1 should also show the length of the polyA tails on human transcript with and without infection (not just Table S1).

3. in the method section (and the start of the result section), the authors describe using A549 cells for CoV2 infection but say that the CoV2 data was collected from publicly available datasets and do not describe CoV2 infections. At the start of the result section, they also don’t mention the SLK cells used for KSHV infection, the HEK293 cells used for EBV infection, the ARPE-19 cells used for HSV2. All together, this comes out as confusing as to which infection they performed, which cells they extracted RNA from etc. Please streamline. And include all cell lines in Fig1A for accurate comparison.

4. line 280: typo in sgRNA – it should be sgmRNA

5. line 308-309: rephrase: it is unclear what a “polyA tail of differing length” means.

6. for consistency, fig 3A/3B should show the “modal” tail length on the graph

Reviewer #2: (No Response)

**********

PLOS authors have the option to publish the peer review history of their article (what does this mean?). If published, this will include your full peer review and any attached files.

If you choose “no”, your identity will remain anonymous but your review may still be made public.

**Do you want your identity to be public for this peer review?**  For information about this choice, including consent withdrawal, please see our Privacy Policy.

Reviewer #1: No

Reviewer #2: No

**Figure resubmission:**

-->While revising your submission, we strongly recommend that you use PLOS’s NAAS tool (https://ngplosjournals.pagemajik.ai/artanalysis) to test your figure files. NAAS can convert your figure files to the TIFF file type and meet basic requirements (such as print size, resolution), or provide you with a report on issues that do not meet our requirements and that NAAS cannot fix.

After uploading your figures to PLOS’s NAAS tool - https://ngplosjournals.pagemajik.ai/artanalysis, NAAS will process the files provided and display the results in the "Uploaded Files" section of the page as the processing is complete. If the uploaded figures meet our requirements (or NAAS is able to fix the files to meet our requirements), the figure will be marked as "fixed" above. If NAAS is unable to fix the files, a red "failed" label will appear above. When NAAS has confirmed that the figure files meet our requirements, please download the file via the download option, and include these NAAS processed figure files when submitting your revised manuscript.

**Reproducibility:**

To enhance the reproducibility of your results, we recommend that authors of applicable studies deposit laboratory protocols in protocols.io, where a protocol can be assigned its own identifier (DOI) such that it can be cited independently in the future. Additionally, PLOS ONE offers an option to publish peer-reviewed clinical study protocols. Read more information on sharing protocols at https://plos.org/protocols?utm_medium=editorial-email&utm_source=authorletters&utm_campaign=protocols

# Author comment: Author response to Decision Letter 1

**DOI:** 10.1371/journal.ppat.1014341.r002

5 May 2026

Attachment Submitted filename: Response to reviewer comments.pdf

# Peer review: Decision Letter 1

**DOI:** 10.1371/journal.ppat.1014341.r003

**Authors:** John Karijolich

26 May 2026

PPATHOGENS-D-26-00002R1

Extended poly(A) tails are a shared feature of herpesvirus mRNAs

PLOS Pathogens

Dear Dr. Depledge,

Thank you for submitting your manuscript to PLOS Pathogens. After careful consideration, we feel that it has merit but does not fully meet PLOS Pathogens's publication criteria as it currently stands. Therefore, we invite you to submit a revised version of the manuscript that addresses the points raised during the review process.

Please submit your revised manuscript by Jul 25 2026 11:59PM. If you will need more time than this to complete your revisions, please reply to this message or contact the journal office at plospathogens@plos.org. When you're ready to submit your revision, log on to https://www.editorialmanager.com/ppathogens/ and select the 'Submissions Needing Revision' folder to locate your manuscript file.

Please include the following items when submitting your revised manuscript:

* A letter that responds to each point raised by the editor and reviewer(s). You should upload this letter as a separate file labeled 'Response to Reviewers'. This file does not need to include responses to any formatting updates and technical items listed in the 'Journal Requirements' section below.

* A marked-up copy of your manuscript that highlights changes made to the original version. You should upload this as a separate file labeled 'Revised Manuscript with Track Changes'.

* An unmarked version of your revised paper without tracked changes. You should upload this as a separate file labeled 'Manuscript'.

If you would like to make changes to your financial disclosure, competing interests statement, or data availability statement, please make these updates within the submission form at the time of resubmission. Guidelines for resubmitting your figure files are available below the reviewer comments at the end of this letter.

As the corresponding author, your ORCID iD is verified in the submission system and will appear in the published article. PLOS supports the use of ORCID, and we encourage all coauthors to register for an ORCID iD and use it as well. Please encourage your coauthors to verify their ORCID iD within the submission system before final acceptance, as unverified ORCID iDs will not appear in the published article. Only the individual author can complete the verification step; PLOS staff cannot verify ORCID iDs on behalf of authors.

We look forward to receiving your revised manuscript.

Kind regards,

John Karijolich, Ph.D.

Academic Editor

PLOS Pathogens

Blossom Damania

Section Editor

PLOS Pathogens

Sumita Bhaduri-McIntosh

Editor-in-Chief

PLOS Pathogens

orcid.org/0000-0003-2946-9497

Michael Malim

Editor-in-Chief

PLOS Pathogens

orcid.org/0000-0002-7699-2064

**Additional Editor Comments**

We were able to obtain comments from one of the original reviewers for this revised manuscript. As you will see, the reviewer continues to raise some concerns. After careful editorial evaluation, we believe these concerns can be addressed through a minor revision.

In particular, the authors should carefully temper the language throughout the manuscript to avoid overinterpretation of the findings. In addition, validation of the knockdown presented in Figure 7 will be important for strengthening the conclusions.

**Journal Requirements:**

If the reviewer comments include a recommendation to cite specific previously published works, please review and evaluate these publications to determine whether they are relevant and should be cited. There is no requirement to cite these works unless the editor has indicated otherwise.

1. Your current Financial Disclosure states, “DPD is supported by a German Centre for Infection Research (DZIF, https://www.dzif.de/en) Associate Professorship and also receives funding from the Deutsche Forschungsgemeinschaft (DFG, https://www.dfg.de/en, German Research Foundation) under Germany's Excellence Strategy - EXC 2155 - project number 390874280 (https://www.resist-cluster.de/en/). HB is supported by the Medical Research Council (MRC, https://www.ukri.org/councils/mrc/) (MR/Z505523/1) and the Academy of Medical Sciences (https://acmedsci.ac.uk/)(SBF008\1027). EL, PC, and CJ were supported by the Hannover Biomedical Research School (HBRS) and the Center for Infection Biology (ZIB). CJ was funded by the Deutsche Forschungsgemeinschaft (DFG, German Research Foundation) project number 405772731 (GZ: VI 761/1-1). KK was funded by the Deutsche Forschungsgemeinschaft (DFG, German Research Foundation)—SFB 900/3—project number 158989968 to AV-B (TPB9) (https://www.mh-hannover.de/sfb900.html). ACW is supported by NIAID (https://www.niaid.nih.gov/) grants R01AI176335 and R01-AI170583. REW, WW, and MF are supported by the MRC (MR/Z505444/1). None of the funders had any role in the study design, data collection and analysis, decision to publish, or preparation of the manuscript.”.

However, your funding information on the submission form indicates is missing the funder name "EL, PC, and CJ were supported by the Hannover Biomedical Research School (HBRS) and the Center for Infection Biology (ZIB) and KK was funded by the Deutsche Forschungsgemeinschaft (DFG, German Research Foundation)—SFB 900/3". Please indicate by return email the full and correct funding information for your study and confirm the order in which funding contributions should appear. Please be sure to indicate whether the funders played any role in the study design, data collection and analysis, decision to publish, or preparation of the manuscript.

2) PLOS now requires that authors provide the original uncropped and unadjusted images underlying all blot or gel results reported in a submission’s figures or Supporting Information files. This policy and the journal’s other requirements for blot/gel reporting and figure preparation are described in detail at https://journals.plos.org/plosone/s/figures#loc-blot-and-gel-reporting-requirements and https://journals.plos.org/plosone/s/figures#loc-preparing-figures-from-image-files.

When you submit your revised manuscript, please ensure that your figures adhere fully to these guidelines and provide the original underlying images for all blot or gel data reported in your submission. See the following link for instructions on providing the original image data: https://journals.plos.org/plosone/s/figures#loc-original-images-for-blots-and-gels.

In your cover letter, please note whether your blot/gel image data are in Supporting Information or posted at a public data repository, provide the repository URL if relevant, and provide specific details as to which raw blot/gel images, if any, are not available. Email us at customercare@plos.org if you have any questions.

**Reviewers' Comments:**

Reviewer's Responses to Questions

**Part I - Summary**

Please use this section to discuss strengths/weaknesses of study, novelty/significance, general execution and scholarship.

Reviewer #1: I thank the authors for addressing many of the reviewers’ comments and for revising several of the previously overstated or speculative interpretations. As noted in the initial review, and similarly emphasized by Reviewer 2, the study nevertheless remains largely descriptive and still lacks mechanistic insight. I still believe that this study is interesting and of broad interest, yet several of the conclusions continue to extend beyond what is directly supported by the experimental evidence presented. Additional experimental validation would strengthen the robustness of the conclusions make it more suitable for this journal.

**********

**Part II – Major Issues: Key Experiments Required for Acceptance**

Please use this section to detail the key new experiments or modifications of existing experiments that should be absolutely required to validate study conclusions.

Generally, there should be no more than 3 such required experiments or major modifications for a "Major Revision" recommendation. If more than 3 experiments are necessary to validate the study conclusions, then you are encouraged to recommend "Reject".

Reviewer #1: Specific comments:

- PABPC expression across cell types:The authors have now included PABPC expression analyses in three cell lines and explain that additional cell lines could not be assessed. However, in their response, they propose that shorter EBV poly(A) tail lengths could correlate with higher PABPC expression levels. This is again speculative, as no experimental data directly support such link. In the manuscript, the authors state that this “could explain their slightly lower overall modal mRNA poly(A) tail length,” but this interpretation would require functional validation (for example, PABPC or CCR4 depletion experiments). Moreover, given that only three cell lines were analyzed, the evidence is insufficient to establish a meaningful correlation between PABPC expression and poly(A) tail length at this stage.

- Conclusions regarding cytoplasmic localization (lines 398–401):The authors state that “translation or cytoplasmic localisation appear not to be prerequisites for extended poly(A) tail lengths on herpesviral transcripts.” I remain concerned that this conclusion is too broad relative to the data presented. Both Reviewer 2 and I raised this issue during the initial review, yet no additional experiments were included to directly support this claim (cell fractionation for example). As written, the statement reads as a general mechanistic conclusion that is not sufficiently substantiated by the current data.

- CNOT1 silencing experiments (Figure 7):It appears that the CNOT1 silencing experiments were performed only in duplicate. The authors should include a western blot demonstrating the efficiency of the knockdown. In addition, it is unclear whether the data presented in Figure 7 represent a single experiment or the average of biological replicates. The figure legend should clarify this point. The legend also does not clearly indicate which cell type was used for the silencing experiments, and the Methods section does not include how the knockdown was performed.

**********

**Part III – Minor Issues: Editorial and Data Presentation Modifications**

Please use this section for editorial suggestions as well as relatively minor modifications of existing data that would enhance clarity.

Reviewer #1: (No Response)

**********

PLOS authors have the option to publish the peer review history of their article (what does this mean?). If published, this will include your full peer review and any attached files.

If you choose “no”, your identity will remain anonymous but your review may still be made public.

**Do you want your identity to be public for this peer review?**  For information about this choice, including consent withdrawal, please see our Privacy Policy.

Reviewer #1: No

[NOTE: If reviewer comments were submitted as an attachment file, they will be attached to this email and accessible via the submission site. Please log into your account, locate the manuscript record, and check for the action link "View Attachments". If this link does not appear, there are no attachment files.]

**Figure resubmission:**

-->While revising your submission, we strongly recommend that you use PLOS’s NAAS tool (https://ngplosjournals.pagemajik.ai/artanalysis) to test your figure files. NAAS can convert your figure files to the TIFF file type and meet basic requirements (such as print size, resolution), or provide you with a report on issues that do not meet our requirements and that NAAS cannot fix.

After uploading your figures to PLOS’s NAAS tool - https://ngplosjournals.pagemajik.ai/artanalysis, NAAS will process the files provided and display the results in the "Uploaded Files" section of the page as the processing is complete. If the uploaded figures meet our requirements (or NAAS is able to fix the files to meet our requirements), the figure will be marked as "fixed" above. If NAAS is unable to fix the files, a red "failed" label will appear above. When NAAS has confirmed that the figure files meet our requirements, please download the file via the download option, and include these NAAS processed figure files when submitting your revised manuscript.

**Reproducibility:**

To enhance the reproducibility of your results, we recommend that authors of applicable studies deposit laboratory protocols in protocols.io, where a protocol can be assigned its own identifier (DOI) such that it can be cited independently in the future. Additionally, PLOS ONE offers an option to publish peer-reviewed clinical study protocols. Read more information on sharing protocols at https://plos.org/protocols?utm_medium=editorial-email&utm_source=authorletters&utm_campaign=protocols

# Author comment: Author response to Decision Letter 2

**DOI:** 10.1371/journal.ppat.1014341.r004

1 Jun 2026

Attachment Submitted filename: Response_to_reviewer_comments_auresp_2.pdf

# Editor report: Decision Letter 2

**DOI:** 10.1371/journal.ppat.1014341.r005

**Authors:** John Karijolich

2 Jun 2026

Dear Depledge,

We are pleased to inform you that your manuscript 'Extended poly(A) tails are a shared feature of herpesvirus mRNAs' has been provisionally accepted for publication in PLOS Pathogens.

Before your manuscript can be formally accepted you will need to complete some formatting changes, which you will receive in a follow up email. A member of our team will be in touch with a set of requests.

Please note that your manuscript will not be scheduled for publication until you have made the required changes, so a swift response is appreciated.

IMPORTANT: The editorial review process is now complete. PLOS will only permit corrections to spelling, formatting or significant scientific errors from this point onwards. Requests for major changes, or any which affect the scientific understanding of your work, will cause delays to the publication date of your manuscript.

Should you, your institution's press office or the journal office choose to press release your paper, you will automatically be opted out of early publication. We ask that you notify us now if you or your institution is planning to press release the article. All press must be co-ordinated with PLOS.

Thank you again for supporting Open Access publishing; we are looking forward to publishing your work in PLOS Pathogens.

Best regards,

John Karijolich, Ph.D.

Academic Editor

PLOS Pathogens

Blossom Damania

Section Editor

PLOS Pathogens

Sumita Bhaduri-McIntosh

Editor-in-Chief

PLOS Pathogens

orcid.org/0000-0003-2946-9497

Michael Malim

Editor-in-Chief

PLOS Pathogens

orcid.org/0000-0002-7699-2064

***********************************************************

Reviewer Comments (if any, and for reference):

# Editor report: Acceptance letter

**DOI:** 10.1371/journal.ppat.1014341.r006

**Authors:** John Karijolich

Dear Depledge,

We are delighted to inform you that your manuscript, "Extended poly(A) tails are a shared feature of herpesvirus mRNAs," has been formally accepted for publication in PLOS Pathogens.

We have now passed your article onto the PLOS Production Department who will complete the rest of the pre-publication process. All authors will receive a confirmation email upon publication.

The corresponding author will soon be receiving a typeset proof for review, to ensure errors have not been introduced during production. Please review the PDF proof of your manuscript carefully, as this is the last chance to correct any scientific or type-setting errors. Please note that major changes, or those which affect the scientific understanding of the work, will likely cause delays to the publication date of your manuscript. Note: Proofs for Front Matter articles (Pearls, Reviews, Opinions, etc...) are generated on a different schedule and may not be made available as quickly.

Soon after your final files are uploaded, the early version of your manuscript, if you opted to have an early version of your article, will be published online. The date of the early version will be your article's publication date. The final article will be published to the same URL, and all versions of the paper will be accessible to readers.

For Research Articles, you will receive an invoice from PLOS for your publication fee after your manuscript has reached the completed accept phase. If you receive an email requesting payment before acceptance or for any other service, this may be a phishing scheme. Learn how to identify phishing emails and protect your accounts at https://explore.plos.org/phishing.

Thank you again for supporting open-access publishing; we are looking forward to publishing your work in PLOS Pathogens.

Best regards,

Sumita Bhaduri-McIntosh

Editor-in-Chief

PLOS Pathogens

orcid.org/0000-0003-2946-9497

Michael Malim

Editor-in-Chief

PLOS Pathogens

orcid.org/0000-0002-7699-2064
